# Evaluation of the Scandinavian guidelines for head injuries based on a consecutive series with computed tomography from a Norwegian university hospital

**DOI:** 10.1186/1757-7241-20-62

**Published:** 2012-09-04

**Authors:** Ingrid Haavde Strand, Ole Solheim, Kent Gøran Moen, Anne Vik

**Affiliations:** 1 Department of Radiology and Diagnostic Imaging, St.Olavs University Hospital, Trondheim, N-7006, Norway; 2 Department of Neurosurgery, St.Olavs University Hospital, Trondheim, N-7006, Norway; 3 MI-Lab, Department of Circulation and Medical Imaging, Norwegian University of Science and Technology, Trondheim, N-7006, Norway; 4 Department of Neuroscience, Norwegian University of Science and Technology, Trondheim, N-7006, Norway

**Keywords:** CT scan, Guideline compliance, Minor head injury, Traumatic brain injury

## Abstract

**Background:**

This study prospectively assesses clinical characteristics and management of consecutive minimal, mild and moderate head injury patients referred for CT scans. Compliance with the Scandinavian head injury guidelines and possible reasons for non-compliance is explored.

**Methods:**

From January 16^th^ 2006 to January 15^th^ 2007, 1325 computed tomography (CT) examinations due to minimal, mild or moderate head injury according to the Head Injury Severity Scale (HISS) were carried out at our University Hospital. When ordering a CT scan due to head trauma, physicians were asked to fill out a questionnaire.

**Results:**

Guideline compliance was impossible to assess in 49.5% of all cases. This was due to non-assessable or missing key variables necessary in the decision making algorithm. One or more key variables for HISS classification were not assessable in 34.4% as it was unknown whether there had been loss of consciousness (LOC), duration of LOC was unknown or it was impossible to assess amnesia or focal neurologic deficits. Definite compliance with both CT and admittance recommendations in guidelines was seen in only 31.2%. In 54.2% of patients with minimal head injuries who underwent CT scans, imaging was not necessary according to guidelines. 59.1% of all patients were admitted to hospital, however only 23.7% of these were admitted because of the head-injury alone. Age < 4 years, possible medical cause of injuries, severe headache/nausea or vomiting and the presence of non-traumatic CT findings were independently associated with non-assessable compliance with Scandinavian guidelines. Suspicion of influence of alcohol was inversely associated to non-compliance.

**Conclusions:**

Despite the prospective study design, guideline compliance was not assessable in nearly half of the patients. Patients with isolated head injuries and available and obtainable complete clinical information necessary for guideline-based decision making are not dominating in a head injury population.

## Background

Guidelines for the initial management of minimal, mild or moderate head injuries [1-5] were implemented approximately a decade ago. They provide evidence based algorithms for decision making, including indications for CT examinations, admission for observation or discharge in patients with head injuries. The decision trees vary some but are all based on combinations of a set of clinical and anamnestic factors, such as the Glasgow Coma Scale (GCS) score on arrival, loss of consciousness (LOC), amnesia, vomiting, headache, focal neurologic deficits, intoxication, suspected skull fracture, seizures, dangerous trauma mechanisms, failure to improve, coagulopathy, and/or prior neurosurgery. Sensitivity of detection of patients requiring neurosurgical interventions is for most guidelines close to 100%, but specificity is much lower [6].

Patient management can be affected by numerous factors not taken into consideration in the guidelines, including the distance to hospital, patient co-morbidities and other injuries, limitation of radiology services, availability of hospital beds, patient demands, severity of pain, language barriers, the ability to be observed at home and local transportation logistics. Also, anamnestic and clinical information necessary for guideline based decision making may be unavailable when deciding to order CTs, discharge, or admit for observation. Thus, while guidelines can provide good support for clinical decisions, decision rules are sometimes not directly applicable and not always followed.

Head injury guideline compliance has been sparsely studied [7-9]. A retrospective study from 2003, two years after the implementation of the Scandinavian guidelines, found that overall compliance was only 51%, and that over triage was seen in 69% of patients with minimal injuries. However, since many of the important patients’ characteristics were unavailable, reasons for non-compliance could not be explored [10].

We conducted a prospective study based on all CT-examinations for minimal, mild and moderate head injuries in a one year period in a Norwegian university hospital. We aimed to assess clinical characteristics, management and compliance with Scandinavian guidelines and explore possible reasons for non-compliance.

## Methods

### The study setting and patient referral

St.Olavs Hospital, University Hospital of Trondheim, Norway is a level I trauma centre for 680,000 inhabitants and houses the only neurosurgical department in the region and hence receives all severe and many moderate head injuries from three counties. Additionally, the hospital serves as a local hospital for 275,000 inhabitants. Many minimal and mild head injuries are initially managed by general practitioners either at their offices during day-time, at regional emergency clinics, or on call in the emergency clinic at the hospital. Some patients are referred to the neurosurgical resident on call, before or after CT examinations. Other suspected head injuries are managed directly by resident neurosurgeons, typically patients transported directly by ambulance or helicopter from site of injury. Further, some head injuries are initially managed by the hospital trauma team, when there is suspected or confirmed multi-trauma or high energy trauma, according to the advanced trauma life support (ATLS) guidelines. Additionally, some head injuries may be referred by or admitted to various hospital departments as part of more complex medical settings, e.g. syncope, seizure, intoxication or possible stroke followed by head injury.

### The implementation of guidelines and information about the study

**Figure 1:**
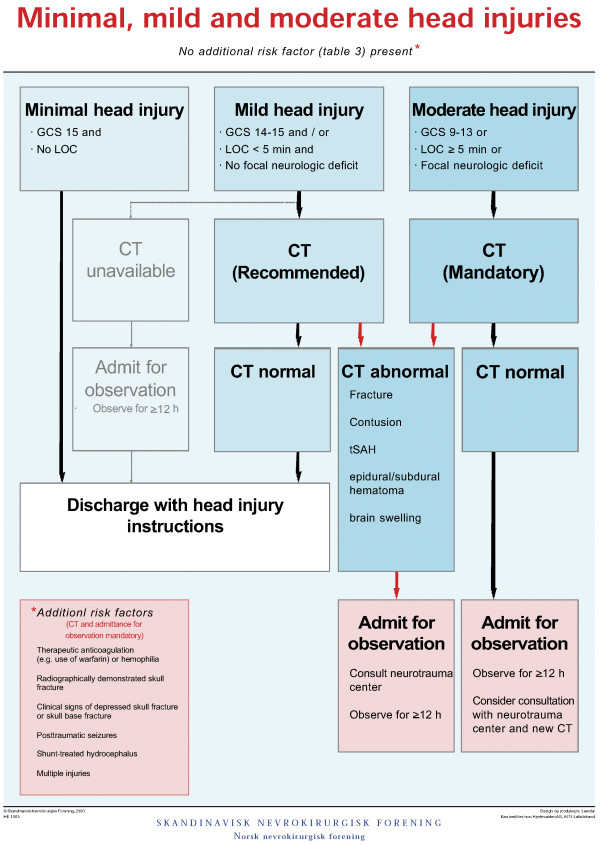
**Decision-making algorithm by the Scandinavian Neurotrauma Committee. **The figure shows the Scandinavian decision-making algorithm for the management of minimal, mild and moderate head injuries.

The Scandinavian guidelines [5] were implemented after a national campaign in 2000, and the decision algorithm is based on the Head Injury Severity Scale (HISS) [11] and the presence of additional risk factors (Figure 1). Prior to and at the beginning of the inclusion period, the first author (IHS) reminded the various in-hospital departments involved (neurosurgical, ENT, neurology, internal medicine, surgical departments, general practitioners’ emergency clinic and radiology department) about the Scandinavian guidelines and informed about the prospective study protocol and its questionnaire.

### The inclusion process

**Figure 2:**
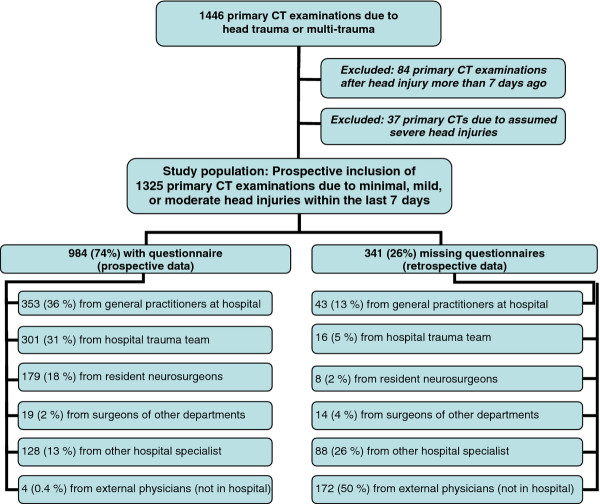
**The inclusion and exclusion process. **The inclusion and exclusion process leading to a study population of 1325 primary CT examinations after recent minimal, mild, or moderate head trauma is shown.

During the one-year period from January 16^th^ 2006 to January 15^th^ 2007, 1446 primary CT scans due to head trauma or multi-trauma were carried out (Figure 2). After exclusion of assumed severe head injuries and CTs due to head trauma that was more than 7 days ago, 1325 primary CTs of patients with recent minimal, mild or moderate head injuries were included in the present study. 396 (30%) of the CT scans were ordered by general practitioners at the emergency clinic in the hospital, 317 (24%) were requested by the hospital trauma team due to suspected or confirmed multi-trauma or high energy trauma,187 (14%) were ordered by resident neurosurgeons, 33 (3%) by surgeons from other departments, 216 (16%) by other hospital specialists, and 176 (13%) by general practitioners outside the hospital.

### The questionnaire and study variables

When ordering head CT examinations due to trauma, in-hospital physicians were asked to fill out a questionnaire. The primary response was 708/1152 (62%), but increased to 984 (85%) after a reminder. In addition to the 169 missing questionnaires from in-hospital physicians, questionnaire based data was missing from 173 CT examinations ordered by general practitioners not working in the hospital, thus unaware of the study and its questionnaire. In cases with missing or incomplete questionnaires we (IHS) attempted to retrieve missing variables from hospital medical records and radiology order forms. However, in many cases it was impossible to obtain key variables through this retrospective review, and such variables are categorized as *missing*. If data was not obtainable due to the patient’s condition or lack of reliable witnesses, some questionnaire items were prospectively reported or retrospectively classified as *unknown* or *not possible to assess.* Patient management was classified as either compliant with guidelines, non-compliant with guidelines or impossible to assess according to this point-by-point list:

Compliant (y/n)

∘ Isolated minimal, mild or moderate head injury managed according to guidelines

Non-compliant (y/n)

∘ Minimal head injury, no additional risk factors, but CT performed

∘ Isolated mild head injury, normal CT, no risk factors and admittance to hospital

∘ Moderate head injury without admittance to hospital

∘ Minimal or mild injury, risk factors, without admittance to hospital

∘ Mild head injury within the last 12 h, normal CT, living alone, without admission to hospital

∘ Minimal or mild head injury within the last 12 hours, traumatic CT findings but not admittance to hospital

Impossible to assess (y/n)

∘ Admittance due to other injuries or medical causes

∘ Unknown/Non-assessable key-variables

∘ Missing data

### Time of assessment

Since aiming to investigate guideline compliance, only the situation prior to ordering the CT examination was assessed. Thus, classification into to the HISS categories and other clinical variables reflects the last known status before ordering CT examinations. Likewise, when reviewing patient hospital records, we attempted to explore the situation prior to ordering the CT. Radiology reports in 43 (3.2%) of the CT examinations were later changed, but we utilized the initial image descriptions in the present study, since these formed basis for clinical decisions at the time.

### Statistics

Data was analyzed in SPSS version 19 for Windows. Q-Q-plots were used to test for normal distribution. Central tendencies are presented as medians when skewed. Mann–Whitney-U test was used for significance testing in ordinal data with skewed distributions. Chi-square test was used for significance testing in contingency tables and Fisher’s exact test was used where sample sizes were less than 5. Binary regression analyses were performed, and predictor variables with p-values less than 0.1 were included in multivariable regression models. Statistical significance level was set to p < 0.05.

### Ethics

The study was approved by the Regional Ethical Committee for Health Region Mid-Norway and the Norwegian Social Science Data Services. Inclusion of patient data without informed consent was approved by the Norwegian Directorate of Health.

## Results

### General characteristics and classification according to HISS

Age, sex, injury mechanism and time of CT for the 1325 patients are presented in Table 1. While 1.8% were infants/toddlers, 22.6% were elderly (≥ 65 years).

**Table 1:** Some characteristics of all the 1325 included patients

**General characteristics**	**N = 1325**
**Gender**	
Male	754 (56.9%)
**Age**	
Median [Range]	35 [0-100]
Infant/toddler (0-3)	24 (1.8)
Children (4-15)	169 (12.8%)
Adolescents/young adults (16-20)	160 (12.1%)
Adults (21-64)	673 (50.8%)
Elderly (≥65)	299 (22.6%)
**Trauma mechanism**	
Falls	688 (51.9%)
Traffic accidents	349 (26.3%)
Violent assaults	107 (8.1%)
Other*	140 (10.6%)
Unknown/missing data	41 (3.1%)
**Time from trauma to CT scan**	
Median	2.6 hours
<12 hours	785 (59.2%)
>12 hours	94 (7.1%)
Unknown/missing data	446 (33.7%)
**Time of CT scan**	
Weekday (Mon-Fri, 08-15)	343 (25.9%)
Weekday evening (Mon-Fri 15-22)	288 (21.7%)
Weekday night (Mon-Thu 22-08)	143 (10.8%)
Weekend/Holiday (Fri -Mon 15-08)	551 (41.6%)

*Includes head traumas without falling or violence and/or possible medical cause of trauma (e.g. syncope, epilepsy, cardiac arrest etc.).

We were unable to classify 365 (27.5%) of the included patients according to the HISS-criteria, either due to non-assessable key-variables or missing key-variables. Questionnaires were missing in 237 (64.9%) of these, most often when CT orders were placed by general practitioners outside the hospital. To explore the possibility of imputation of missing variables, we compared patients who were classifiable according to the HISS criteria with non-classified patients (Table 2). The two groups differed significantly for all tested variables. Imputation of missing variables was therefore not performed.

**Table 2:** Comparison of patients with missing or non-assessable variables for HISS classification and patients with complete data

**Characteristics**	**Classified according to HISS N = 960**	**Not classified according to HISS N = 365**	**p-values**
**Questionnaire responders**			
Yes	855 (89.1%)	128 (35.1%)	<0.001
**Time of CT examination**			
Weekend/Holiday	446 (46.5%)	105 (28.2%)	<0.001
**Trauma mechanism**			
Injury while being hospitalized	5 (0.5%)	29 (8.0%)	<0.001
**Male**	581 (60.5%)	173 (47.4%)	<0.001
**Age**			
Median	30 years	52 years	<0.001
**Clinical affiliation of CT ordering doctor**			<0.001
GP emergency clinic (in hospital)	322 (33.5%)	74 (20.3%)	
Neurosurgical department	167 (17.4%)	20 (5.5%)	
Other hospital department	102 (10.6%)	147 (40.3%)	<0.001
Trauma team	309 (32.2%)	8 (2.2%)	
External GP (not in hospital)	60 (6.3%)	116 (31.8%)	
**CT findings**			
Traumatic findings (%)	91 (9.5%)	12 (3.3%)	<0.001
**Admittance to hospital?**			
Yes	593 (61.8%)	196 (53.7)	0.007
**Neurosurgical intervention?**			
Yes	12 (1.3%)	0 (0%)	0.027

### Non-assessable or unknown key variables for HISS classification

At presentation, one or more variables potentially crucial for HISS classification were frequently unknown or non-assessable due to the patients’ condition or lack of witness observations (Table 3). In 456 (34.4%) of all 1325 patients, 382 (83.8%) of whom had available questionnaire data, either GCS score was non-assessable (intubation), the presence or the duration of LOC was either unknown or not assessable, it was not possible to assess if the patient had posttraumatic amnesia, or it was not possible to assess if the patient had focal neurologic deficits. Both infants/toddlers (<4 years) and elderly (≥ 65 years) were significantly (p < 0.001) more seldom classifiable into HISS categories, despite available questionnaires. Assumed focal neurologic deficits before the CT examination were reported in 12.6% of elderly patients (≥65 years), as compared to 4.0% of the younger patients (p < 0.001).

**Table 3:** Reported clinical findings and symptoms important for HISS classification at the clinical examination before CT

**Clinical findings and symptoms at presentation**	**Minimal injury**	**Mild injury**	**Moderate injury**	**Missing data or not classifiable**	**Overall**
	**N = 275**	**N = 525**	**N = 160**	**N = 365**	**N = 1325**
**Glasgow Coma Scale score**					
15	271 (98.5%)	379 (72.2%)	64 (40.0%)	129 (35.3%)	843 (63.6%)
14	N/A	140 (26.7%)	15 (9.4%)	24 (6.6%)	179 (13.5%)
9-13	N/A	N/A	68 (42.5%)	13 (3.6%)	81 (6.1%)
Intubated/sedated	2 (0.7%)	2 (0.4%)	7 (4.4%)	4 (1.1%)	15 (1.1%)
Missing data	2 (0.7%)	4 (0.8%)	6 (3.8%)	195 (53.4%)	207 (15.6%)
**Loss of consciousness (LOC)?**					
No	236 (85.8%)	64 (12.2%)	28 (17.5%)	69 (18.9%)	397 (30.0%)
Yes	N/A	306 (58.3%)	70 (43.8%)	122 (33.4%)	499 (37.7%)
<5 minutes	N/A	243 (46.3%)	23 (14.4%)	62 (17.0%)	329 (24.8%)
≥5 minutes	N/A	N/A	38 (23.8%)	15 (4.1%)	56 (4.2%)
Unknown length of LOC Ω	N/A	63 (12.0%)	9 (5.6%)	45 (12.3%)	114 (8.6%)
Unknown if LOC Ω	35 (12.7%)	148 (28.2%)	39 (24.4%)	58 (15.9%)	280 (21.1%)
Missing data	4 (1.5%)	7 (1.3%)	23 (14.4%)	116 (31.8%)	149 (11.2%)
**Posttraumatic amnesia?**					
No, n (%)	254 (92.4%)	99 (18.9%)	33 (20.6%)	66 (18.1%)	452 (34.1%)
Yes, n (%)	0 (0%)	394 (75.1%)	62 (38.8%)	98 (26.8%)	555 (41.9%)
Impossible to assess	16 (5.8%)	22 (0.4%)	32 (20.0%)	38 (10.4%)	107 (8.1%)
Missing data	5 (1.8%)	10 (1.9%)	33 (20.6%)	163 (44.7%)	211 (15.9%)
**Assumed focal neurologic deficits?**					
No	260 (94.5%)	492 (93.7%)	73 (45.6%)	256 (70.1%)	1081 (81.6%)
Yes	0 (0%)	0 (0%)	57 (35.6%)	10 (2.7%)	67 (5.1%)
Impossible to assess	6 (2.2%)	18 (3.4%)	17 (10.6%)	18 (4.9%)	59 (4.5%)
Missing data	9 (3.3%)	15 (2.9%)	13 (8.1%)	81 (22.2%)	118 (8.9%)

Ω Loss of consciousness, or duration was impossible to assess due to lack of witnesses and uncertain anamnestic information.

### Additional risk factors from the Scandinavian guidelines

Before the CT examination, 28.2% of all patients had one or more “additional risk factors” according to the decision algorithm (Figure 1, Table 4). If including patients with later radiological diagnosed cranial fracture, this percentage increases to 29.5%.

**Table 4:** Reported risk factors and other variables important for decision making according to Scandinavian guidelines

**Risk factors and other clinical findings and symptoms**	**Minimal injury**	**Mild injury**	**Moderate injury**	**Missing data or not classifiable**	**Overall**
	**N = 275**	**N = 525**	**N = 160**	**N = 365**	**N = 1325**
**Risk factors from the Scandinavian Guidelines** Ω					
· Therapeutic anticoagulation or hemophilia	12 (4.4)	12 (2.3%)	7 (4.4%)	26 (7.1%)	57 (4.3%)
· Clinical suspicion of impression fracture or skull base fracture	10 (3.6%)	23 (4.4%)	8 (5.0%)	13 (3.6%)	54 (4.1%)
· Posttraumatic epileptic seizure	1 (0.4%)	5 (1.0%)	5 (3.1%)	5 (1.4%)	16 (1.2%)
· Shunt due to hydrocephalus	1 (0.4%)	1 (0.2%)	1 (0.6%)	0 (0%)	3 (0.2%)
· Multi-traumatized patient #	112 (40.7%)	105 (20.0%)	42 (26.3%)	9 (2.5%)	268 (20.2%)
**Additional patient characteristics**					
· Severe headache, nausea or vomiting	61 (22.2%)	168 (32.0%)	25 (15.6%)	114 (31.2%)	368 (27.8%)
· Use of anti-platelet drugs	17 (6.2%)	20 (3.8%)	20 (12.5%)	51 (14.0%)	108 (8.2%)
· Living alone					
Yes	32 (11.6%)	73 (13.9%)	33 (20.6%)	66 (18.1%)	204 (15.4%)
Missing data	4 (1.5%)	8 (1.5%)	11 (6.9%)	79 (21.6%)	102 (7.7%)
**Clinical suspicion of alcohol/drug intoxication at presentation**					
· No	209 (76.0%)	354 (67.4%)	92 (57.5%)	121 (33.2%)	776 (58.6%)
· Yes	27 (9.8%)	128 (24.4%)	50 (31.3%)	25 (6.9%)	230 (17.4%)
· Unknown/missing data	39 (14.2%)	43 (8.2%)	18 (11.3%)	219 (60.0%)	319 (24.1%)

Ω Radiological cranial fracture is also an additional risk factor.

# Questionnaire marked for multi-traumatized patient or CT of multiple organs ordered by hospital trauma team (trauma CT), or multiple radiological examinations of different organs ordered by treating physicians.

Severe headache, nausea or vomiting was observed in 27.8%, a clinical suspicion of alcohol or drug intoxication was reported in 17.4% and at least 15.4% of all patients were living alone.

### CT findings and management after CT examination

As seen in Table 5, intracranial traumatic CT findings were primarily described in 6.3% and cranial fractures in 2.9%. Non-traumatic pathological findings were described in 20.2%. Only 0.9% of the 1325 patients underwent a neurosurgical intervention, most often patients with moderate head injuries.

**Table 5:** CT image findings (initial description) and patient management

**CT findings and management after CT examination**	**Minimal injury**	**Mild injury**	**Moderate injury**	**Missing data or not classifiable**	**Overall**
	**N = 275**	**N = 525**	**N = 160**	**N = 365**	**N = 1325**
**CT findings**					
Normal CT *	218 (79.3%)	423 (80.6%)	88 (55.0%)	241 (66.0%)	970 (73.2%)
Intracranial traumatic findings	10 (3.6%)	36 (6.9%)	30 (18.8%)	7 (1.9%)	83 (6.3%)
Epidural hematoma	1 (0.4%)	2 (0.4%)	4 (2.5%)	0 (0%)	7 (0.5%)
Subdural hematoma	3 (1.1%)	12 (2.3%)	15 (9,4%)	1 (0.3%)	31 (2.3%)
SAH/intraventricular hemorrhage	2 (0.7%)	12 (2.3%)	12 (7.5%)	3 (0.8%)	29 (2.2%)
Contusion hematoma	4 (1.5%)	15 (2.9%)	17 (10.6%)	5 (1.4%)	41 (3.1%)
Diffuse cerebral edema	1 (0.4%)	1 (0.2%)	5 (3.1%)	0 (0%)	7 (0.5%)
Pneumocephalus	1 (0.4%)	8 (1.5%)	7 (4.4%)	0 (0%)	16 (1.2%)
Cranial fracture(s)	4 (1.5%)	17 (3.2%)	12 (7.5%)	5 (1.4%)	38 (2.9%)
With impression	1 (0.4%)	2 (0.4%)	2 (1.3%)	0 (0%)	5 (0.4%)
Without impression	1 (0.4%)	7 (1.3%)	6 (3.8%)	3 (0.8%)	17 (1.3%)
Skull base fracture	2 (0.7%)	10 (1.9%)	7 (4.4%)	2 (0.5%)	21 (1.6%)
Non-traumatic pathological findings	45 (16.4%)	64 (12.2%)	44 (27.5%)	114 (31.2%)	267 (20.2%)
**Admission to hospital**					
Number of admissions	188 (68.4%)	285 (54.3%)	120 (75.0%)	196 (53.7%)	789 (59.6%)
Median length of stay (days)	1.0	0.5	1.5	0.5	0.5
Range length of stay (days)	0.0 – 52.0	0.0 - 98.0	0.0 - 108.0	0.0 - 60.0	0.0 – 108.0
Admission to hospital due to:					
Head injury alone	30 (10.9%)	103 (19.6%)	37 (23.1%)	16 (4.4%)	186 (14.0%)
Other causes than injuries	3 (1.1%)	7 (1.3%)	7 (4.4%)	68 (18.6%)	85 (6.4%)
Other injuries than the head injury	86 (31.3%)	35 (6.7%)	6 (3.8%)	16 (4.4%)	143 (10.8%)
Combinations	68 (24.7%)	140 (26.7%)	70 (43.8%)	95 (26.0%)	373 (28.2%)
Unknown cause/missing data	1 (0.4%)	0 (0%)	0 (0.0%)	1 (0.3%)	2 (0.2%)
Not admitted	87 (31.6%)	240 (45.7%)	40 (25.0%)	169 (46.3%)	536 (40.5%)
**Neurosurgical intervention**	3 (1.1%)	1 (0.2%)	8 (5.0%)	0 (0%)	12 (0.9%)
Intracranial pressure monitoring	0 (0%)	1 (0.2%)	4 (2.5%)	0 (0%)	5 (0.4%)
Reposition of cranial fracture(s)	1 (0.4%)	0 (0%)	2 (1.3%	0 (0%)	3 (0.2%)
External ventricle drain	0 (0%)	0 (0%)	0 (0%)	0 (0%)	0 (0%)
Craniotomy	1 (0.4%)	0 (0%)	2 (1.3%)	0 (0%)	3 (0.2%)
Burr hole-procedure§	1 (0.4%)	0 (0%)	0 (0%)	0 (0%)	1 (0.1%)
**In-house-mortality**	3 (1.1%)	1 (0.2%)	2 (1.3%)	8 (2.2%)	14 (1.1%)

*Except extra-cranial hematoma or swelling.

§ Evacuation of chronic subdural hematoma.

789 (59.5%) of the 1325 who underwent CT scans were admitted to hospital. Among patients who were admitted, combined causes were most common (47.3%). Only 186 (23.6%) of admitted patients were admitted because of head injury alone.

### Compliance with Scandinavian guidelines

Overall compliance with guidelines was impossible to assess in 49.5% of all 1325 cases (Table 6). This was due to non-assessable or missing key variables for HISS classification or patients who were admitted due to other injuries/other medical causes, or when it was unknown if patients were living alone. Non-compliance was seen in 19.3%, either due to unnecessary CT scans (11.2%), violation of the admission recommendations, or both. Thus, definite compliance with the Scandinavian guidelines was seen in 31.2%. However, overall compliance was 61.7% for the 669 assessable patients. 54.2% of patients with minimal head injuries had overtriage with CT scans.

**Table 6:** Compliance with Scandinavian guidelines for minimal, mild and moderate head injuries

**Compliance to Scandinavian guidelines**	**Minimal injury**	**Mild injury**	**Moderate injury**	**Missing data or not classifiable**	**Overall**
	**N = 275**	**N = 525**	**N = 160**	**N = 365**	**N = 1325**
Compliance with CT recommendation					
Yes	126 (45.8%)	525 (100%)	160 (100%)	78 (21.4%)	889 (67.1%)
No	149 (54.2%)	0 (0%)	0 (0%)	0 (0%)	149 (11.2%)
Impossible to assess	0 (0%)	0 (0%)	0 (0%)	287 (78.6%)	287 (21.7%)
Compliance with admittance recommendation					
Yes					
No	102 (37.1%)	290 (55.2%)	82 (51.2%)	120 (32.9%)	594 (44.8%)
Impossible to assess or admitted due	16 (5.8%)	68 (13.0%)	25 (15.6%)	9 (2.5%)	118 (8.9%)
to other reasons than head injury	157 (57.1%)	167 (31.8%)	53 (33.1%)	236 (64.7%)	613 (46.3%)
Compliance with both CT and admittance recommendations					
Yes					
No	12 (4.4%)	289 (55.0%)	81 (50.6%)	31 (8.5%)	413 (31.2%)
Impossible to assess	152 (55.3%)	69 (13.1%)	26 (16.2%)	9 (2.5%)	256 (19.3%)

### Predictors of non-assessable compliance with CT guidelines (i.e. when are guidelines difficult to apply?)

Age < 4 years, possible medical cause of injuries, severe headache/nausea or vomiting, and the presence of non-traumatic CT findings were independently associated with non-assessable compliance in the multivariable analyses (Table 7). Use of antiplatelet drugs tended also to be an independent predictor.

**Table 7:** Possible predictor variables associated with non-assessable guidline compliance

**Variables in the regression model**	**Unadjusted Odds ratio**	**95% CI**	**p-value**	**Adjusted Odds ratio**	**95% CI**	**p-value**
Age < 4 years	3.1	1.4 to 7.1	0.006	25.6	9.1 to 72.0	<0.001
Age ≥ 65 years	2.7	2.0 to 3.6	<0.001	1.8	0.8 to 4.4	0.180
Gender : female	1.7	1.3 to 2.2	<0.001	1.2	0.6 to 2.1	0.654
Suspicion of alcohol/drug influence	0.4	0.3 to 0.8	0.003	0.8	0.3 to 1.9	0.602
Severe headache, nausea or vomiting	1.9	1.4 to 2.5	<0.001	2.1	1.1 to 4.0	0.034
Possible medical cause of injury*	41.3	24.2 to 70.5	<0.001	234.6	108.5 to 507.3	<0.001
Use of anti-platelet drugs, e.g. acetylsalicylic acid	3.4	2.2 to 5.0	<0.0|01	2.5	1.0 to 6.5	0.052
Non-traumatic CT findings	2.3	1.7 to 3.1	<0.001	3.3	1.5 to 7.3	0.003

*Such as syncope, epilepsy, cardiac arrest etc.

### Predictors of non-compliance with guidelines

Suspicion of influence by alcohol or drugs was inversely associated with non-compliance with guidelines (O.R. 0.4, p = 0.003). There was also a trend towards a positive association with severe nausea/headache or vomiting (p = 0.100). Neither gender, toddlers (age < 4), elderly (age ≥65), trauma mechanism, clinical affiliation of CT ordering physician, CT-examination at night (22:30–08:00), non-traumatic CT findings, posttraumatic amnesia, living alone, nor physical signs of head injury were associated with non-compliance with guidelines in univariable analyses.

### Sensitivity of Scandinavian guidelines

One 72 year old patient with minimal head injury after a fall from own height and no risk factors who underwent a CT examination after 95 minutes, non-compliant to guidelines, deteriorated in the CT lab and underwent surgery due to an acute subdural hematoma (60 ml). Thus, the sensitivity for detecting lesions of neurosurgical significance was 11/12 (92%) (Table 5). Twelve of the 103 patients with traumatic CT findings underwent overtriage with CT, according to the Scandinavian guidelines, while compliance with CT guidelines was impossible to assess in another 10 patients with traumatic findings. Sensitivity for detecting any traumatic finding based on the Scandinavian guidelines may therefore be estimated to be between 78% and 88%.

## Discussion

In this large study in patients assumed to have minimal, mild and moderate head injuries before CT examinations, there was much heterogeneity in terms of patient characteristics, co-morbidity, and health care management. Despite the prospective study design in a university hospital setting, definite compliance with guidelines was observed in only one third of the patients. More striking; for nearly half of the patients, compliance was not assessable. Key factors in the guideline decision algorithm were frequently simply not obstainable, including the presence and duration of LOC, posttraumatic amnesia and focal neurological deficits. It was further observed that admission due to the head injury alone was accounting for only one quarter of the hospital admissions. This reflects a limitation in the direct applicability of decision guidelines in many patients referred for CT scans due to head injuries.

### Compliance with Scandinavian guidelines

In Norway and other countries where emergency medicine is not defined as an own specialty with separate departments, head injuries are primarily managed by a range of departments and specialists, including general practitioners outside the hospital, perhaps increasing the difficulty of enforcing guidelines. We found that 11.2% of CT scans, more than half in patients with minimal head injuries, were unnecessary, while one fifth were either admitted to hospital or discharged in contradiction to the recommendations in the Scandinavian guidelines. Definite compliance with guidelines was therefore only observed in one third of all patients. Among assessable patients, overall guideline compliance was 61.7%. In comparison, two other studies have reported the overall compliance with Scandinavian guidelines to be 51% and 60.5%, respectively [7,12]. A recent study demonstrated an almost perfect compliance for adults requiring head CT according to the NICE guidelines, but a much lower compliance in children [9]. However, oddly compliance was always assessable in these studies, even retrospectively [7,9,12].

### Predictors of non-compliance

We found that guidelines seemed more difficult to apply in toddlers, patients with medical causes of injuries, severe nausea/headache or vomiting or presence of non-traumatic CT findings. These findings are not surprising and are clinical factors not covered in the Scandinavian guidelines. Headache and/or vomiting are, however, symptoms included in the decision algorithm of several other guidelines [1,3,9].

Also, we found that compliance may be better when there is suspicion of influence by alcohol or drugs, whereas the other studied factors were not predictors for non-compliance. In contrast, a recent retrospective study reported that physicians' guideline compliance is not affected by the patients' alcohol consumption [12].

### The co-morbidity of head injuries

The co-morbidity of head injuries and the fact that they often also occur in more complex medical settings, can make it difficult to directly apply guidelines in many cases. Barely a fourth of the admitted patients were admitted due to the head injury alone. Thus, isolated minimal or mild head injuries in patients without other injuries or diseases, not living alone, are perhaps more seldom than expected. The presence of possible “additional risk factors” (as defined by the guidelines) when examining the patient in the ER, was frequent (28.2%), and since at least 15.4% lived alone, observation at home and the recommended nightly wake-up-checks are also sometimes problematic. We observed that presentation with assumed focal neurologic deficits and thereby a possible moderate head injury, was reported significantly more often in the elderly, perhaps illustrating possible assessment difficulties if there is neurologic comorbidity or even normal aging*.*

### Sensitivity of guidelines

As only 0.9% underwent brain or skull surgery after CT examination; 0.3% if excluding the moderate injuries, outcome is usually good, often regardless of compliance with management guidelines. Also to be remembered, guidelines are not perfect and clinical judgment is still necessary. We found a sensitivity of 92% for detection of findings in need of surgery. Although the Scandinavian guidelines perform quite well in comparison with other guidelines [13], a recent systematic review concluded that the Canadian CT Head Rule is more widely validated and has demonstrated the most consistent results [6]. Still to be remembered, although guidelines seem to perform quite well in various validation studies [14], everyday practice may be different. The inclusion and exclusion criteria of studies behind the guidelines and their respective validation studies may affect external validity of results. For example, in a recent validation study of 6936 head injury patients, only 52% met the study inclusion criteria [14]. This potentially affects the generalizability of results to everyday settings. Imputation of missing variables or conducting complete data analyses, as often seen in the decision rule literature, seems dubious due to significant differences between groups as demonstrated in our study.

### Unnecessary CT scans and impact on health economics

In the pre-guideline era, some authors recommended routine CT as a screening tool, with selective admission being based on CT findings [8,15-18], while others recommended routine admission with or without CT scanning [19,20]. Also, there used to be considerable inter-hospital variations in patient management [21,22]. A controlled study reported that early CT and discharge is cheaper and at least as effective as hospital admission in mild injuries [23]. Liberal use of CT scanning based on a high sensitivity decision rule may therefore be both effective and cost-saving, since the cost of CT scanning is small compared to costs of caring for patients with head injury worsened by delayed treatment [24]. Still, unnecessary CT examinations should be avoided both to save health personnel resources and avoid unnecessary radiation, concerns and morbidification. Head injury guidelines can theoretically reduce unnecessary CT scans, without compromising patient safety [25,26]. However, an earlier randomized controlled study showed that rates of CT imaging were not reduced [27], and they may even be increased after guideline implementation [28]. A recent study among CT ordering physicians report that “fear of missing a traumatic intracranial lesion” may explain many “unnecessary CT scans”, despite knowledge about management guidelines [29]. As seen in our study, compliance with guidelines is far from perfect, and over triage with unnecessary CT was observed in more than half of the classifiable minimal injuries. Hypothetical health economic analyses that assume that guidelines are or can be followed may therefore be overly optimistic and simplified.

### Time of assessment and external validity

We recorded the clinical information obtained before the CT examination in this study. However, as an indication of the dynamics in such patient evaluations, we found that 25% of patients that before CT were assumed to have moderate head injuries were not even admitted to hospital. Risk stratification and recommendations in most management guidelines, is usually based on the clinical situation at one point of time. Clinical evaluation of a head injury patient is nevertheless a continuous process based on the anamnestic information available from the time of injury along with history or observations of improvements or deteriorations over time, before and after seeking medical attention. Anamnestic information, witness information, symptoms and level of consciousness will change, and most often improve over time.

Our findings are based on the review of CT examinations in a Norwegian university hospital with a neurosurgical department. These findings may therefore not directly be extrapolated to other, patient selections, regions, countries, health care systems, or treatment guidelines. Even so, the frequent inability to apply management guidelines directly due to missing key information is probably a universal problem that has received little attention.

### Study strengths and limitations

The prospective and rigid questionnaire-based evaluation of physicians’ diagnostic considerations prior to ordering CT scans, the unselected consecutive patient population, along with the magnitude of this study makes it among the most rigorous effort to date to evaluate head injury guideline compliance in everyday clinical practice. Inclusion was nevertheless based only on patients who underwent CT examinations. We have therefore no information on under triage with CT, i.e. direct discharge without CT examination.

To ideally study compliance with and performance of management guidelines, at least two prospective study points would be preferable; one before CT scans, and one at admittance or discharge from hospital. This could probably increase the compliance in the group of assumed moderate injury in this study, since most of them would have been reclassified to the mild, and some even to the minimal group. Our study also suffered from the inability to stratify all patients into the risk groups according to HISS. This is partial a response-rate problem, but also reflects the challenge of categorizing heterogeneous trauma patients in a dynamic state with frequent confounding factors and limited anamnestic and clinical information available.

## Conclusions

Despite the prospective study design, guideline compliance was not assessable in half of the patients. Important key variables for guideline based decision making were often not obtainable at the time of CT referral, and admission due to the head injury alone was quite uncommon. Definite compliance with guidelines was observed in only about one third of all patients and over triage with CT scan was observed in more than half of minimal injuries. The factors of importance for risk stratification according to guidelines were most often difficult to assess in infants/toddlers, in patients with possible medical cause of injuries, severe headache/nausea or vomiting and the presence of non-traumatic CT findings. We still believe that decision rules can be an important clinical support in many cases and can ensure a framework for common practice with minimal risks of under triage. However, the frequent challenge with non-obtainable key clinical variables should be considered when updating guidelines.

## Competing interests

The authors declare that they have no competing interests.

## Authors’ contributions

HIS participated in the design of the study, collected the data and performed statistical analyses. OS performed the statistical analyses and drafted the manuscript. KGM performed statistical analyses, made figures and helped to draft the manuscript. AV participated in the design of the study, and helped to draft the manuscript. All authors read and approved the final manuscript.
